# Predicting Microbiome Metabolism and Interactions through Integrating Multidisciplinary Principles

**DOI:** 10.1128/mSystems.00768-21

**Published:** 2021-10-05

**Authors:** Caleb M. Schmidt, Parsa Ghadermazi, Siu Hung Joshua Chan

**Affiliations:** a Advanced Pattern Analysis & Countermeasures Group, Boulder, Colorado, USA; b Department of Systems Engineering, Colorado State University, Fort Collins, Colorado, USA; c Department of Chemical and Biological Engineering, Colorado State University, Fort Collins, Colorado, USA

**Keywords:** metabolic modeling, microbial communities, microbiome

## Abstract

In this Commentary, we will discuss some of the current trends and challenges in modeling microbiome metabolism. A focus will be the state of the art in the integration of metabolic networks, ecological and evolutionary principles, and spatiotemporal considerations, followed by envisioning integrated frameworks incorporating different principles and data to generate predictive models in the future.

## COMMENTARY

**FIG 1:**
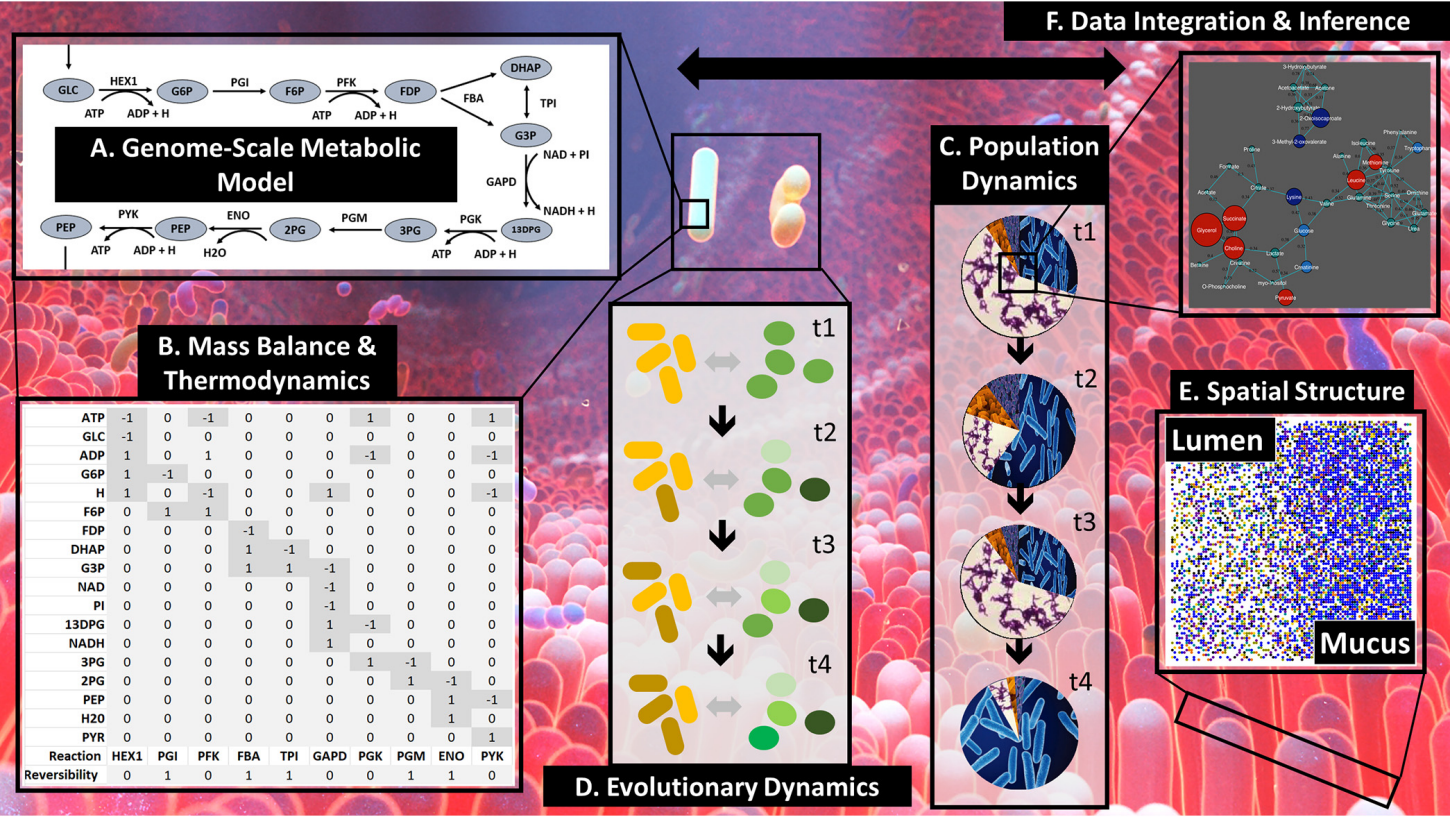
Integrating genome-scale metabolic models (A and B), population (C) and evolutionary (D) dynamics, spatial structures (E) (image adapted from *PLoS Computational Biology* [19]), and omics data (F) for predicting microbiome metabolism.

The past 2 decades have seen significant increases in the technical ability of metabolic models to more accurately represent the underlying metabolic dynamics in biological systems. What started as models of simple organisms, representing only a subset of their genes (1), has now evolved into multicompartmental human models (2). This positive trend in experimental and modeling capability has also allowed for the construction of multiorganism, microbiome systems with significant nuance. This Commentary describes the state of the art in metabolic modeling (especially as it relates to microbiomes) and entertains a selection of considerations that, we believe, are highly relevant to continued progress in the field (Fig. 1).

## GENOME-SCALE METABOLIC NETWORK

A hallmark approach in metabolic modeling is genome-scale metabolic modeling (GEM). The goal of a GEM is to depict the metabolic structure of a cell using the genome sequence as a blueprint, allowing for a complete mapping of reactions in a cell. On top of this blueprint is layered the known metabolic reactions, gene data, and biochemical data to generate a curated reaction network (Fig. 1A). Within this framework, the cell system is framed as a stochiometric matrix (Fig. 1B). By applying steady-state mass balance across the system and constraining the reaction directionality based on thermodynamics, a solvable system of linear equations can be optimized for a given cellular objective function, e.g., biomass production, to obtain a set of flux distributions across all reactions in the cell. This forms the basis of flux balance analysis (FBA) (3).

The GEM approach described above has shown significant success in human systems (e.g., references 4 and 5). A sex-specific, organ-resolved GEM that incorporates ∼80,000 metabolic reactions with relevant anatomical and physiological data was recently published (2). For the microbial counterpart, GEMs have provided a mechanistic framework connecting the chemical environment to microbial metabolism. GEMs can accurately predict metabolic phenotypes and engineering strategies for rewiring microbial metabolism for various biomedical and biotechnological applications, e.g., biochemical production. One important recent advance is the development of automated reconstruction tools (6), such as KBase and CarveMe, that allow for massive rapid model reconstruction in large-scale studies. For example, AGORA2 generates draft models for >7,000 strains using KBase (7). It is currently the largest gut microbial model collection and can be used to simulate drug metabolism affected by human gut microbes. The history and state of the art are reviewed elsewhere in greater detail (8). When extending to modeling microbiomes, however, there are still significant limitations and challenges, e.g., insufficient taxonomic resolution and biochemical knowledge for nonmodel organisms, computational scalability from single-organism to community models, and more general evolutionary principles governing communities needed. These are all related, and here we will look into this from the perspective of integrating ecological and evolutionary principles.

## ECOLOGICAL AND EVOLUTIONARY PRINCIPLES

A decade of constructing microbial community GEMs has made it clear that different model structures and algorithms have different assumptions and answer different questions. In this context, there are three relevant questions that must be addressed. The first is whether to use a computationally efficient supraorganism model (microbiome as a single organism) or a dynamically more accurate community model (microbiome as multiple-assembled organism genomes). A second important consideration is whether static versus dynamic simulations will be used (discussed below) (9). A third relevant question is whether to use a single-level or multilevel objective optimization approach. Each of these considerations involves different ecological and evolutionary principles and assumptions (Fig. 1C and D). We will take a deeper look at the second and third questions.

### Static, single-level frameworks.

Single-level approaches optimize across the entire community (e.g., by assuming group selection and optimizing the total fitness of the community, instead of individuals). Evolutionary biologists could be critical of this approach because it bypasses selection at the individual level. Indeed, FBA omits certain stable interactions within communities and can be prone to unrealistic altruistic predictions (10). Community FBA was proposed to implement an equal growth rate constraint across the community. This framework allows for a population steady-state assumption to be followed in stable communities (11). We have proposed a reformulated algorithm, called SteadyCom, that is able to perform flux variability analysis (FVA) and demonstrated its scalability to large community models. Through this approach, we were able to predict the gut microbiota relative abundance profile given an average American diet (10).

### Static, multilevel frameworks.

In contrast, multilevel approaches try to find solutions in which each individual member of a community maximizes its fitness function. This approach is closer to the accepted theory of natural selection/adaptation but elevates the computational demand. OptCom was the first bilevel optimization paradigm to consider both individual and community fitness (12). Building on this concept, we have recently proposed a new bilevel algorithm, called NECom, to formally implement the concept of Nash equilibrium (NE) in microbial metabolic models (13). NE is a concept from evolutionary game theory in which no individual microbe can increase its fitness unilaterally and thus each microbe is maximizing its fitness function simultaneously. We showed the superior predictive capability of NECom in a large-scale data set of mutualistic alga-yeast cocultures compared to FBA, with a 70% improvement in prediction of member growth rates under ∼500 uptake conditions (13).

### Evolutionary dynamic frameworks.

Dynamic flux balance analysis (dFBA) juxtaposes the steady-state assumptions of FBA (14). dFBA models the time-dependent extracellular metabolite concentrations by solving FBA-embedded differential equations. In each time step, uptake constraints in FBA are updated to solve for new exchange fluxes. However, we recently showed that dFBA cannot predict the coevolution of community members, e.g., the complementation of amino acid auxotrophic Escherichia coli pairs, because each can act only upon metabolites present in their environments (13). That is, a microbe is unable to “know” what other microbes in the system are capable of and act accordingly. Therefore, coevolved, stable, mutualistic interactions favored by natural selection may be missing.

This dFBA deficiency is partially addressed by a recent algorithm based also on NE by Zomorrodi and Segrè (15) in which mutants from the same species with alternative metabolic strategies are predefined and simulated until an NE is reached. By solving only linear programs, the algorithm is computationally efficient but requires predefined strategies. In contrast, the NECom algorithm predicts NE using network structures alone but is computationally demanding (solving bilevel programs). An evolutionary dynamic framework with evolving strategies for individual microbes similar to that used in reference 16 could have the advantages of both.

### Spatiotemporal dynamics.

dFBA has achieved success in ground truthing models to experimental data and allowed for introducing spatiotemporal dynamics to microbial ecosystems. This is possible through the addition of differential equations for spatial dynamics and/or compartmentalization based on spatial structures to produce a realistic picture. We were able to capture the observed differential relative abundance profiles of microbiota radially, from the intestinal lumen to the mucosal layer, and longitudinally, along the intestinal tract, by proposing a dynamic model of the mucosal cell layer shedding into the lumen directing flow into the succeeding intestinal section and combining SteadyCom and dFBA (17). Explicit spatial dynamics further improves the resolution. COMETS incorporates concentration-based diffusion dynamics to model the global spatiotemporal dynamics. It is able to predict nonintuitive competition-mutualism dynamics that match experimental results (18). BacArena (19) (Fig. 1E) and IndiMeSH (20) are also important examples of platforms that allow investigators to spatially resolve microbial populations and molecular signatures in different matrices.

## DATA-DRIVEN INTERACTION INFERENCE AND HYPOTHESIS TESTING

Algorithms have been developed to integrate omics data into the metabolic network structure to identify interactions and test mechanistic hypotheses (Fig. 1F). In our previous work, we have tested the validity of mechanistic hypotheses, e.g., bile salt hydrolase activity in the *Clostridia* causing observed gut microbial metabolomic change after drug treatment (21), and tested which microbes are likely to be responsible for the observed fecal metabolomic changes in infants during initial microbial colonization (22). More sophisticated techniques, such as machine learning (ML) algorithms, have helped in generating, maintaining, and integrating experimental data into models to help infer the phenome and other information about the system. For example, ML has been successful in supporting investigators to better annotate genomes, fill gaps in GEMs, choose constraints for models, and integrate omics data into GEMs (23). Increases in the scale and complexity of data only support the continuation of this trend to better understand and integrate data.

## THE FUTURE—INTEGRATION OF PRINCIPLES AND HETEROGENEOUS DATA SET

We have seen how GEMs can capture biological observations with very few parameters and meanwhile how machine learning can improve the accuracy of predictions based on observed data. We believe that seamless integration of the aforementioned (ecological, evolutionary, and spatiotemporal) principles in a systematic modeling framework with parameters learned through rigorous model inference techniques will connect theory and data to enhance the usefulness of GEMs for studying complex microbiomes. This process of integration includes gathering heterogenous data sets where myriad supervised and unsupervised methods can be used to concatenate, transform, or model to create algorithm-based models (24). For example, one can train uptake kinetic parameters and resource allocation constraints in a community dFBA model that is evolutionarily stable and cross-validated by concentration, relative abundance data, and other omics data. Further, these models can be used as an analytical framework as well as to suggest microbial/genetic/nutritional/spatial perturbations that alter the evolutionary fate of the community.

Within the set of experimental techniques to generate these data sets, there are two flavors: bottom-up versus top-down. As an alternative to the more commonly used bottom-up synthetic community approach, top-down approaches to study smaller communities of a complex microbiome are emerging. A recent study deconstructed the soil microbiome into functional modules through targeted enrichment of desired metabolic traits. It allows for investigating a community with significantly less complexity and drawing mechanistic conclusions (25). Another promising approach is to culture the “unculturable” organism in a microbiome using microfluidic droplets as localized, nanoliter-sized bioreactors. By encapsulating organisms from human fecal samples this way, metagenome-assembled genomes of uncharacterized gut commensals with high resolution can be recovered (26). New modeling tools should be ready to integrate these data to gain new insights into the microbiome.
